# The Impact of Temperature on 24-Hour Movement Behaviors among Chinese Freshmen Students

**DOI:** 10.3390/ijerph20064970

**Published:** 2023-03-11

**Authors:** Hongjun Yu, Yiling Song, Yangyang Wang, Xiaoxin Wang, Haoxuan Li, Xiaolu Feng, Miao Yu

**Affiliations:** 1 Department of Physical Education, Tsinghua University, Beijing 100084, China; 2 Department of Sports Science, College of Education, Zhejiang University, Hangzhou 310058, China; 3 Renmin University of China Libraries, Beijing 100872, China

**Keywords:** temperature, physical activity, sedentary behavior, sleep duration, youth

## Abstract

Background: Human populations worldwide have experienced substantial climate change issues. Gaps in scientific literature remain regarding the relationship between temperature and 24-hour movement behavior among people. The purpose of this study is to examine the impact of temperature on 24-hour movement behavior including physical activity (PA), sedentary behavior (SB) and sleep duration among university students living in Beijing, China. Methods: We conducted follow-up health surveys on 44,693 freshmen students enrolled at Tsinghua University from 2012 to 2018. PA and SB were measured by using the short version of the International Physical Activity Questionnaire (IPAQ-s); sleep duration was estimated by using The Pittsburgh Sleep Quality Index (CPSQI). Corresponding temperature data measured by the Beijing Meteorological Service were collected to include average daily temperature from the nearest weather station to Tsinghua university. The data were analyzed using linear individual fixed-effect regressions. Results: An increase in temperature (temperature range 2.29–28.73 °C) by 1 °C was associated with an increase in 0.66 weekly minutes of vigorous physical activity (VPA) (95% confidence interval [CI] = 0.49, 0.82), an increase in 0.56 weekly minutes of moderate physical activity (MPA)(95% CI = 0.32, 0.79), an increase in 1.21 weekly minutes of moderate to vigorous physical activity (MVPA) (95% CI = 0.90, 1.53), an increase in 0.55 weekly minutes of walking (95% CI = 0.31, 0.78), an increase in 1.76 weekly minutes of total PA (95% CI = 1.35, 2.17), and a reduction in 1.60 weekly minutes of sleeping (95% CI = −2.09, −1.11). There was no significant correlation between temperature and sedentary behavior among participants. Conclusions: Temperature was significantly positively correlated with physical activity levels in the Chinese freshmen students, and significantly negatively correlated with sleep duration. Replication of this study is warranted among various populations within China. The evidence of this novel study focused on understanding the relationship between climate change and 24-hour movement behaviors among people for developing effective adaptation strategies to climate change to improve people’s health behavior. This study has important implications for future study, as knowledge of the impact of temperature on movement behavior may help in the interpretation of their results and translate into improving people’s health behavior.

## 1. Introduction

Climate change poses a great threat to human lives and health in a variety of ways and has become a concern in global health [1]. Studies have shown that climate change has direct or indirect effects on human health [2]. For instance, changes in extreme temperatures can lead to increased morbidity and mortality [3], as well as exposure to toxic substances and degraded air quality [4], all of which affect human health. Even climate change can affect people’s mental health [5]. The health problems caused by climate change have become one of the most important public health problems [6].

At present, physical inactivity is a global public health problem and is the fourth leading cause of death globally [7]. Physical activity (PA) is any bodily movement produced by skeletal muscle contraction that causes energy expenditure to exceed the resting metabolic rate [8]. Regular PA is associated with a reduced risk of all-cause mortality, cardiovascular disease, type 2 diabetes, many cancers, as well as better mental health and quality of life [9,10,11]. Public health guidelines recommend that university students have at least 150 min of moderate physical activity (MPA) or 75 min of vigorous physical activity (VPA) per week [12,13,14]. Sedentary behavior (SB) is a conscious activity characterized by low energy expenditure and performed in a sitting or reclining position, such as watching TV, reading, or driving [15]. Moreover, there is evidence that SB is also a risk factor for health [16]. For example, too much SB may impair cognitive function in the brain [17]. In addition, sleep duration plays a vital role in social, physical, psychological and cognitive health, and it is also an important part of overall health and well-being [18].

PA, SB, and sleep duration are the main movement behaviors that occur in individuals within 24 hours, which are referred to as 24-hour movement behaviors [19]. Appropriate 24-hour movement behavior has important implications for individual health and disease prevention. Therefore, the 24-hour movement behavior of individuals deserves our attention. With global average temperatures likely to be 2 °C above pre-industrial levels by the end of the 21st century [20], understanding the impact of climate change on 24-hour movement behaviors is important. A recent review has reported that climate change affects individuals’ 24-hour movement behaviors [21]. Researchers need to further explore the impact of climate change in order to propose targeted countermeasures for the improvement of human health behavior. For instance, an individual’s physical activity behavior is affected by a variety of factors [22], such as social relationships, education, and climate change. Among the climate change factors that affect physical activity, temperature plays an important role [23,24,25,26].

The natural environment includes many factors that affect physical activity, such as seasons and weather [23]. Previous literature has reported that ambient temperature has a potential impact on physical activity [24]. A cross-sectional study from Norway showed that adolescents had higher odds of meeting physical activity recommendations in spring compared to winter, meaning that a higher temperature was associated with higher levels of physical activity in adolescents [27]. Moreover, one study found that adults were more likely to participate in walking for recreation in summer, autumn and spring compared to winter; more likely to participate in walking for transportation in autumn; and more likely to participate in MPA in summer. The likelihood of achieving adequate MPA was significantly greater in summer, autumn and spring [28]. Another study reported that physical activity volume and more moderate to vigorous physical activity (MVPA) of participants in summer were greater than those in winter, and noted that physical activity volume and MVPA of participants were positively correlated with temperature [29]. Furthermore, a study from Canada showed that Canadians had an average total daily energy expenditure of 31.0% higher in summer than in winter, and were 86% more likely to be active in recreational physical activity in summer than in winter [30]. Ho et al. [31] recently reported that high temperature (between 32.6 °C and 36.5 °C) was associated with decreases of 800–1500 daily steps compared to optimal temperature (between 16 °C and 19.3 °C) among Chinese adults. A recent systematic review and meta-analysis showed that, in children and adolescents, higher temperature was associated with MVPA, while lower temperature was associated with more SB [32]. In addition, a systematic review has shown that ambient temperature also affects an individual’s sleep duration and quality [33]. Research with American teenagers also showed that, when the temperature increased from −13.3 °C to 8.3 °C, the midpoint time of sleep moved to an earlier time, and then when the temperature increased from 8.3 °C to 31.7 °C, the midpoint time of sleep moved to a later time, which showed that the midpoint time of sleep will also be affected by temperature [34].

Despite the aforementioned work, three major gaps in the previous studies remain. First, although there are currently some studies on seasonal climate change and individual health behaviors, there is a lack of studies on the impact of temperature on individual 24-hour movement behavior. Second, there are only a few studies with large cohort samples. Third, studies that consider PA, SB and sleep duration at temperatures among different seasons are lacking. With continued greenhouse gas emissions and global warming due to urban development, we need to consider combining temperature management strategies with PA interventions to potentially promote comfortable and safe PA [35]. The freshmen are in a transitional phase from adolescence to adulthood, a critical time for developing healthy lifestyles and healthy behaviors. Meanwhile, compared to high school, college is a new living environment for freshmen, and their learning style and lifestyle have changed dramatically. Freshmen are experiencing this change and will have more autonomy in choosing their lives and studies, and freshmen are more susceptible to the influence of the outside environment during this period. Therefore, the purpose of this study is to evaluate the impact of temperature on 24-h movement behavior among Chinese freshmen students and to provide a theoretical basis for relevant departments to formulate environmental intervention policies and measures. For example, campus environmental interventions and educational guidance can be used to enable freshmen students to adapt to their new environment while developing healthy behavioral habits. We hypothesized that temperature was positively correlated with PA in Chinese freshmen students, and negatively correlated with SB and sleep duration.

## 2. Methods

### 2.1. Participants

Data from this study were obtained from a paper–pencil-based follow-up health survey that all freshmen at Tsinghua University are asked to fill out. Survey participation was voluntary, and all participants signed a consent form. The study was approved by the Tsinghua University Institutional Review Board (IRB#2012534001). The survey is designed to evaluate the participants’ health behavior, health conditions, and the detailed descriptions of the questionnaire content, implementation procedures, and member cohorts have been published and available elsewhere [36,37]. Briefly, the faculty administered the survey in a class, and all of the freshmen completed the survey within a health education class. In the survey, participants reported their student identification number, which was used to link multiple survey questionnaires completed by the same respondent.

**Figure 1:**
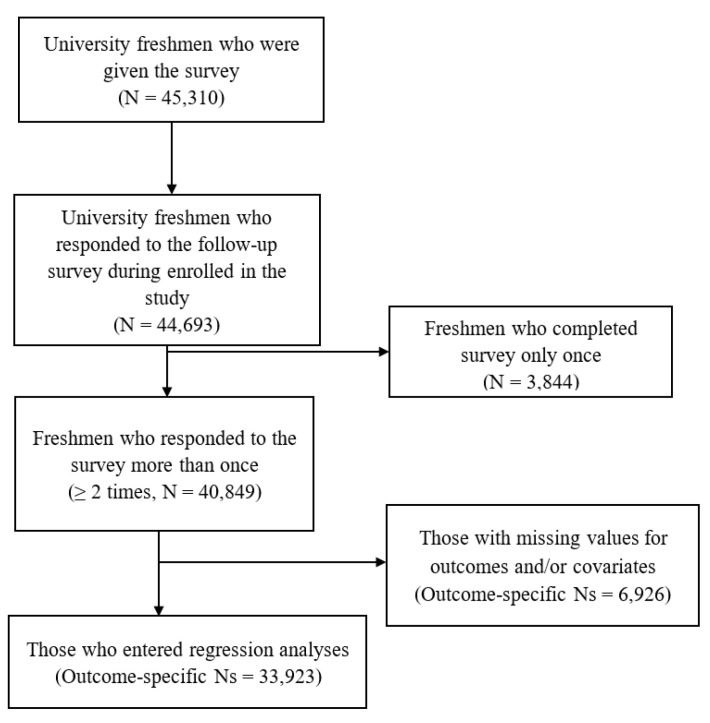
Study Sample Flowchart.

Briefly, the freshmen participants enrolled in a health education class (every other day, Monday to Friday) and completed a faculty-administered paper-based questionnaire. Students handed in the questionnaires at the end of the class. Data on six cohort groups were administered at Tsinghua University: 2012–2013 cohort, 2013–2014 cohort, 2014–2015 cohort, 2015–2016 cohort, 2016–2017 cohort, and 2017–2018 cohort (*n* = 44,693). (See Table 1). The inclusion criteria of this study were as follows: (1) free from diseases or any medical conditions; (2) obtained written informed consent to participate in the study; (3) only freshmen who completed the survey at least two times were included in the analysis. The exclusion criteria of this study were as follows: (1) missing data on physical activity, sedentary behavior or sleep; (2) the participant completed the survey only once. The final sample (*n* = 33,923) had non-missing values for the specific outcome and all covariates (See Figure 1).

### 2.2. Sleep Measurement

We assessed participants’ sleep duration and used The Pittsburgh Sleep Quality Index (CPSQI), which has been validated in China, to measure sleep quality [38].

We modified the question to collect data from the Chinese version of The Pittsburgh Sleep Quality Index (CPSQI): “During the last week, on average, how many hours of sleep did you get per night?” [38,39,40].

### 2.3. Physical Activity Measurement

The short version of the International Physical Activity Questionnaire (IPAQ) was used to measure physical activity. The short version of the IPAQ includes 9 items and has been validated in China [41,42,43]. Data from the short version of the IPAQ provided information on the time spent walking and time spent doing moderate and vigorous intensity activities.

Total minutes of VPA in the last week were calculated based on the answers to two questions adapted from the IPAQ: “During the last seven days, how many days did you do vigorous physical activities, such as aerobics, running, fast bicycling or fast swimming?” and “How much time did you usually spend doing vigorous physical activities on one of those days?”. Total minutes of VPA in the last week were calculated through multiplying daily average number of minutes spent on VPA by its corresponding number of days.

Total minutes of moderate physical activity (MPA) in the last week were calculated based on the answers to two questions adapted from the IPAQ: “During the last seven days, how many days did you do moderate physical activities, such as carrying light loads, bicycling at a regular pace or playing doubles tennis?” and “How much time did you usually spend doing moderate physical activities on one of those days?”. Total minutes of MPA in the last week were calculated through multiplying daily average number of minutes spent on VPA by its corresponding number of days.

Total minutes of walking—light physical activity (LPA)—in the last week were calculated based on the answers to two questions adapted from the IPAQ: “During the last seven days, on how many days did you walk for at least 10 min at a time?” and “How many minutes did you usually spend on one of those days’ walking?”. Total minutes of walking in the last week were calculated through multiplying daily average minutes spent on walking by its corresponding number of days.

Total weekly minutes of MVPA were calculated through multiplying weekly average minutes spent on MPA by weekly average minutes spent doing VPA.

### 2.4. Sedentary Behavior Measurement

The short version of the International Physical Activity Questionnaire (IPAQ), which has been validated in the Chinese population, was used to measure SB [42]. Total hours of SB in the last week were calculated based on the answers to the question adapted from (IPAQ): “During the last 7 days, how much time did you usually spend sitting on a day?” [44] Total hours of SB in the last week were calculated through multiplying daily average hours spent on SB by seven.

### 2.5. Environmental Measures

Meteorological data were obtained on each day that data were collected via the publicly accessible information of the China Meteorological Administration. The nearest weather station to Tsinghua university was identified (Haidian Meteorological Bureau), which is around 4 KM from Tsinghua University. The obtained data were temperature (daily maximum and daily minimum in Celsius degrees), relative humidity (daily average in percentage), average wind speed (m/s), and percentage of rainy days in Beijing, China. These measures were taken seven days before the survey was given. Air pollution data (PM_2.5_) were provided by the Beijing Municipal Ecological Environment Bureau (from 6 December 2013), and the hourly ambient PM_2.5_ concentration data were provided by the Mission China Air Quality Monitoring Program, which was run by the U.S. Department of State (before 6 December 2013, for the 2012–2013 cohort).

### 2.6. Statistical Analyses

Means, SD, and percentages were summarized and compared for characteristics of the overall sample. Continuous variables obtained from follow-up surveys were examined using the one-way repeated measures ANOVA test and t-test. Categorical variables were collated using chi-square tests. Linear individual fixed-effect regressions were performed based on the repeated-measure survey data from the four freshmen cohorts (i.e., 2012–2013, 2013–2014, 2014–2015, 2015–2016, 2016–2017 and 2017–2018). Weekly average minutes of VPA, MPA, and MVPA; daily average hours of sedentary behavior; and daily average hours of sleep in the past week were used for the study’s continuous outcome variable. The key independent variables of the study were average temperature in the past one week. Individual-level time-variant covariates and environmental measures (i.e., average wind speed and percentage of rainy days) and air pollution measure (PM_2.5_) are controlled for the study’s independent variables. An entire sample with both genders, male only, and female only used separate regressions that were conducted for each outcome variable.

We examined the effects of temperature on individual-level physical activity, sedentary behavior and sleep duration outcomes by using linear individual fixed-effect regressions based on the repeated-measure survey data from the six freshmen cohorts (i.e., 2012–2018) at Tsinghua University.

In all regressions, the key independent variable was the standardized, average temperature over the last seven days before the survey. All models were controlled for the individual-level time-variant covariates as well as the environmental variables, including average PM_2.5_ concentration, average wind speed, and percentage of rainy days during the last seven days. Our previous studies [36,37] showed that air pollution had different effects on sedentary behavior and physical activity by gender. Thus, in this study, we examined the effects of temperature on PA, SB and sleep by gender. Each outcome variable was then analyzed by using separate regression and stratified by gender.

The use of fixed effects models is widely accepted for cohort or longitudinal data analysis. Using fixed-effects (FE) models means that we are only interested in analyzing the impact of PA, SB and sleep at varying temperatures. The FE model explores the relationship between temperature and PA, SB and sleep variables within all participants. Each participant has their own individual characteristics that may or may not influence PA, SB and sleep duration (for example, BMI, habits, and personal preferences). When using FE, we assume that something within the individual may impact or bias the PA, SB and sleep outcome variables and that is what we need to control. This is the rationale behind the assumption of the correlation between participants’ error term.

The FE model treats individuals as their own control; all between-person time variation is conditioned out of the model and the model mainly analyzes within-person variation. Furthermore, FE remove the effect of those time-invariant characteristics so we can assess the net relationship between the temperature and PA, SB and sleep. Another important assumption of the FE model is that those time-invariant characteristics are unique to the individual and should not be correlated with other individual characteristics. Potential omitted variable bias due to differences in time-invariant individual characteristics such as genes, gender, ethnicity, habits, and personal preferences was removed through our use of individual fixed-effect regression. Individual fixed-effect regressions could only estimate the effect of a time-variant independent variable due to its exclusive dependence upon within-individual variations in an outcome measure. Therefore, study participant gender, ethnicity, and other time-invariant individual characteristics were not examined.

### 2.7. Individual-Level Covariates

Individual-level time-variant covariates were controlled for in the regression analyses. Continuous variables for age in years; body mass index (BMI; kg/m^2^); self-rated physical health (1–10, poor-excellent); and self-rated mental health (1–10, poor-excellent) were included. Dichotomous variables for current smoking status (non-smokers as the reference group) and drinking status (non-drinkers as the reference group) were additionally measured.

All statistical procedures were performed using Stata 17.0 SE version (StataCorp, College Station, TX, USA). The Eicker-Huber-White sandwich estimator addressed within-individual serial correlations and was used to estimate the standard errors of regression coefficients.

## 3. Results

Table 1 presents the baseline characteristics of the survey participants. A majority of the sample was male participants (67.53%). The mean BMI of participants was 21.39 kg/m^2^ (SD = 3.55). Moreover, only 0.49% of participants were current smokers and 2.58% were current drinkers. The self-rated physical health and self-rated mental health scores had mean values of 5.34 (SD = 2.19) and 6.23 (SD = 2.46), respectively. There were significant differences between males and females in all the above variables (*p* < 0.001).

Table 2 shows the average physical activity, temperature, and other environmental variables in the last seven days before the survey. The average temperature increased from 6.29 °C (SD = 3.45) to 21.43 °C (SD = 5.16), and the weekly total minutes of PA increased from 348.53 min/week (SD = 305.60) to 419.29 min/week (SD = 282.63) within the 2014–2015 cohort. The temperature increased from 25.57 °C (SD = 2.44) to 27.00 °C (SD = 0.82), and the daily total hours of sleeping decreased from 7.34 h/day (SD = 1.01) to 7.03 hr/day (SD = 0.73) within the 2015–2016 cohort. The temperature increased from 5.57 °C (SD = 1.72) to 20.86 °C (SD = 5.18), and the daily total hours of sedentary behavior decreased from 9.45 h/day (SD = 2.86) to 9.22 h/day (SD = 2.90) within the 2016–2017 cohort.

Table 3 reports the estimated effects of temperature on individual-level 24-hour movement behavior outcomes in the past week per day using linear individual fixed-effect regressions. Temperatures were found to be positively associated with total minutes of physical activity in the last week among survey participants. Specifically, an increase in temperature by 1 °C was associated with an increase in weekly minutes of VPA by 0.66 (95% confidence interval [CI] = 0.49, 0.82), an increase in weekly minutes of MPA by 0.56 (95% CI = 0.32, 0.79), an increase in weekly minutes of MVPA by 1.21 (95% CI = 0.90, 1.53), an increase in weekly minutes of walking by 0.55 (95% CI = 0.31, 0.78), and an increase in weekly minutes of total PA by 1.76 (95% CI = 1.35, 2.17). There was no significant correlation between temperature and SB among survey participants. However, temperature was found to be negatively associated with the total minutes of sleep duration in the last week among survey participants. Specifically, an increase in temperature by 1 °C was associated with a reduction in daily minutes of sleeping by 1.60 (95% CI = −2.09, −1.11). There were no significant differences between temperature and PA and sleep duration by gender.

## 4. Discussion

The purpose of this study was to investigate the impact of temperature on 24-hour movement behaviors among Chinese freshmen students. Our study found that temperature had a significant positive correlation with PA behavior in the Chinese freshmen students and a significant negative correlation with sleep duration, which was consistent with some of our hypothesis. However, we did not find a significant negative correlation between temperature and SB.

This study found a significant positive correlation between temperature and individual PA levels in a survey of the Chinese freshmen students, with higher temperature being associated with higher levels of PA, which is consistent with previous findings. A study of 1115 Auckland children showed that for boys, a 10 °C increase in average ambient temperature was associated with a small increase in steps on weekdays and a modest increase in steps on weekends, while the effects for girls were small and unclear [45]. The reasons for this gender-related difference are uncertain, but researchers suggested that outdoor activities, which may be affected by ambient temperature, are more popular among boys than among girls [45]. In addition, a study reported a positive correlation between ambient temperature and physical activity, showing that daily temperature increases strongly predicted increases in daily steps. Regardless of month, every 10 °C increase in temperature was associated with a 2.9% increase in daily steps [46]. Furthermore, a study of 1293 Canadian teenagers showed that for every 10 °C rise in temperature, the time of PA among teenagers would increase by 1% to 2% every day. Moreover, teenagers had less physical activity in winter and more physical activity in warm months [47]. Based on previous studies reporting that levels of PA appear to vary seasonally, the subsequent effects of severe or extreme weather have been identified as barriers to participation in physical activity in different populations [23]. For example, a study from China showed that the average steps of walks for adults in five major Chinese cities at extremely high temperatures(between 32.6 °C and 36.5 °C) dropped by 800 to 1500 steps compared to optimal temperatures (between 16 °C and 19.3 °C) [31]. Obradovich et al. shows that both cold and extremely hot temperatures reduce PA [48]. This is because extreme temperatures may reduce PA due to thermal discomfort, and a cooler climate may have a positive effect on physical activity [35]. In addition, some studies believe that if a person performs PA in a high-temperature environment, it will cause pressure on the individual’s thermoregulatory system, and then the individual may change their behavior [49]. Moreover, a study from the United States [50] reported a non-linear effect of increasing temperature on healthy cycling. Data analysis showed that with increasing temperature, the number of daily cycling hours and distance traveled by individuals increased significantly, but then declined at temperatures above 26–28 °C [50]. Turrisi et al. [29] reported that the natural environment can affect health by promoting or inhibiting PA. Interventions in behavior have to take into account possible weather effects. Extreme weather brought on by climate change may short-circuit health-promoting physical activity and, over time, encourage migration of people in the quest of more hospitable climatic niches. However, there are also some studies that are inconsistent with the findings of this study. For example, a study of 2088 adults in the Arabian Gulf region showed that higher average temperatures and increased humidity were associated with a decrease in the number of steps taken per day [51]. Another study of 40 Chinese adults showed no significant relationship between temperature and PA levels [52]. Inconsistency regarding these finding may be explained by the social or cultural differences among the participants.

Consistent with previous research that there was no significant correlation between temperature and SB among our survey participants [53,54,55,56,57,58], a study on a sample of 740 Hong Kong adolescents reported that there was no significant relationship between temperature and SB during weekdays [57]. Another study from 722 children aged 10–12 years in five countries found that temperature was not significantly associated with SB [58]. However, this study was inconsistent with previous studies in that there was a significant negative association between temperature and SB [59,60,61,62,63]. Katapally et al. reported that that Canadian children were less sedentary in Warm-Wet-Calm weather and more sedentary in Cold-Dry-Windy weather [61]. Only a negative correlation between temperature and sedentary behavior was shown in our study, but it was not statistically significant. A possible explanation for this difference could be that there were differences in the study’s geographic location, region, evaluation method, age, and sample size.

Our study shows a significant negative correlation between temperature and individual sleep duration in Chinese freshmen students, which is consistent with previous studies by scholars. Previous research showed that higher temperatures and longer day lengths are associated with reduced nighttime sleep duration in adolescents [34]. A systematic review found that higher temperatures and extreme weather can affect the quality of sleep in individuals [33]. Moreover, a study from Japan showed that individuals’ sleep duration and sleep quality were affected by seasonal changes, which shows that the longest sleep time is in winter and the shortest is actually in summer [64]. In addition, a study of Danish children showed that compared to spring, children spent 2% longer in sleep in winter [65]. Moreover, a report from the Netherlands showed that adults’ sleep time was extended by 31.8 min/day in winter. Furthermore, previous studies reported that the average sleep time of children in winter was 41 min longer than that in summer, 31 min longer in autumn, and 15 min longer in spring, which may be related to seasonal and temperature changes [66].

To our knowledge, this is the first study to use a longitudinal design to investigate the effect of temperature on 24-h movement behaviors among a large cohort sample size. The repeated measurements in one university ensured variation in temperature elements among Chinese freshmen. However, there are a few major limitations of this study. For example, it used a self-report method to assess PA, SB and sleep duration of the respondents. More precise measurements (e.g., pedometer, connected watches, etc.) are needed for better clarity and reproducibility. There was also a lack of investigation on high temperatures (above 29 °C) and low temperatures (<0 °C). In future studies, we suggest using more objective assessment tools to assess 24-h movement behaviors, such as accelerometers. Furthermore, this study is limited by an important methodological issue, namely the issue of uncertain geography [67], given that people move from one place to another on a daily basis. Therefore, meteorological and air conditions in a fixed geographic setting (in this case, a campus) are not representative of the true environmental exposures experienced by individuals on a daily basis and may further lead to biased scientific findings. We did not use spatial interpolation to get more accurate weather conditions in Tsinghua campus. Thus, the results of this study should be treated with caution. Moreover, all participants were recruited from a convenience sample. Freshmen from one university cannot represent all students in China. In addition, new students’ behaviors may not yet have adjusted to the new environment, and their behaviors such as physical activity and sleeping tend to retain more of the habits they have developed over the years from their previous environment, therefore limiting the generalizability of the study’s findings. Future studies are warranted to produce more generalized estimates.

## 5. Conclusions

This study found that temperature was significantly positively correlated with physical activity levels in Chinese freshmen students, and was significantly negatively correlated with sleep duration. Replication of this study is warranted among various populations within China. This novel study evidence focused on understanding the relationship between climate change and 24-h movement behaviors among people for developing effective adaptation strategies to climate change to improve people’s health behavior. Knowledge of the impact of temperature on movement behavior is likely to be a variable influencing intraindividual 24-h movement-related behavior, which may help interpretation of their results and translate into improving people’s health behavior.

## Figures and Tables

**Table 1:** Baseline characteristics of survey participants.

Characteristics	Male	Female	Total	*p*
Male Sex, *n* (%)	30,181 (67.53)	14,512 (32.47)	44693	
Age (yr), mean (SD)				
2012–2013				
Wave 1	18.12 (0.89)	18.10 (0.76)	18.18 (0.85)	<0.001
Wave 2	18.30 (0.90)	18.17 (0.74)	18.26 (0.86)	<0.001
Wave 3	18.82 (0.99)	18.66 (0.84)	18.77 (0.95)	<0.001
Wave 4	18.93 (0.96)	18.80 (0.81)	18.89 (0.91)	<0.001
2013–2014				
Wave 1	18.28 (0.82)	18.23 (0.75)	18.26 (0.80)	0.1
Wave 2	18.84 (0.81)	18.75 (0.81)	18.81 (0.81)	0.007
2014–2015				
Wave 1	18.10 (0.79)	18.05 (0.65)	18.08 (0.75)	0.08
Wave 2	18.69 (0.84)	18.62 (0.83)	18.66 (0.83)	0.03
Wave 3	18.75 (0.80)	18.71 (0.68)	18.74 (0.76)	0.3
2015–2016				
Wave 1	17.98 (0.72)	17.95 (0.66)	17.97 (0.70)	0.3
Wave 2	18.72 (0.84)	18.63 (0.69)	18.69 (0.79)	0.002
2016–2017				
Wave 1	18.12 (0.77)	18.08 (0.63)	18.11 (0.73)	0.08
Wave 2	18.84 (1.01)	18.76 (0.82)	18.82 (0.96)	0.06
2017–2018				
Wave 1	18.12 (0.78)	18.14 (0.80)	18.13 (0.79)	0.5
Wave 2	18.78 (0.98)	18.75 (0.92)	18.77 (0.97)	0.4
Body mass index, mean (SD)				
BMI (kg/m^2^)	21.90 (3.45)	20.33 (3.50)	21.39 (3.55)	<0.001
Smoking, *n* (%)	187 (0.62)	30 (0.21)	217 (0.49)	<0.001
Drinking *n* (%)	958 (3.17)	197 (1.36)	1155 (2.58)	<0.001
Self-rated physical health, mean (SD)				
Physical health score (1–10)	5.38 (2.22)	5.27 (2.14)	5.34 (2.19)	<0.001
Self-rated mental health, mean (SD)				
Mental health score (1–10)	6.27 (2.46)	6.16 (2.45)	6.23 (2.46)	<0.001
Disease number, mean (SD)	0.51 (0.50)	0.55 (0.54)	0.52 (0.50)	<0.001

Notes: wave 1: the first survey, wave 2: the second repetitive survey, wave 3: the third repetitive survey, wave 4: the fourth repetitive survey.

**Table 2:** Average PA, SB and sleep duration, temperature and other environmental variables in the last seven days before the survey.

Freshmen Cohort	Survey Order	VPA	MPA	MVPA	Walk PA	Total PA	Sedentary Behavior	Sleeping/Day	Temperature (°C)	PM_2.5_ (μg/m^3^)	Wind (m/s)	Rain (%)
2012–2013 cohort ^a^	Sep 17–27	90.05 (106.63)	205.33 (153.79)	295.37 (206.57)	70.41 (113.78)	365.79 (257.68)	8.96 (2.78)	7.09 (0.81)	25.36 (2.87)	72.90 (50.26)	3.23 (0.75)	0.36 (0.51)
	Oct 29–Nov 8	100.72 (89.19)	188.15 (148.56)	288.87 (196.25)	67.89 (111.3)	356.76 (256.06)	9.42 (2.89)	7.06 (0.94)	11.64 (4.20)	68.78 (58.48)	3.55 (0.65)	0.18 (0.41)
	Feb 25–Mar 8	76.18 (97.83)	154.82 (132.17)	231 (184.92)	72.25 (102.73)	303.25 (224.36)	9.23 (2.82)	7.32 (0.84)	12.17 (5.13)	165.13 (131.98)	3.38 (1.00)	0.08 (0.29)
	May 6–16	124.45 (103.86)	176.24 (129.08)	300.69 (190.65)	79.66 (106.95)	380.35 (237.76)	9.24 (2.88)	7.27 (1.01)	28.73 (3.00)	92.88 (70.43)	3.41 (0.44)	0.18 (0.41)
2013–2014 cohort ^b^	Dec 9–15	91.08 (105.08)	163.48 (147.22)	254.56 (200.12)	73.76 (105.34)	328.32 (245.65)	9.33 (2.89)	7.08 (0.90)	5.43 (1.40)	28.75 (15.41)	3.57 (0.67)	0.00 (0.00)
	May 5–11	102.98 (114.05)	164.88 (133.2)	267.86 (197.93)	76.64 (100.11)	344.50 (236.25)	9.28 (2.83)	7.27 (0.96)	20.71 (3.09)	52.8 (21.63)	3.14 (0.24)	0.43 (0.54)
2014–2015 cohort ^c^	Oct 6–12	100.68 (110.54)	194.44 (158.79)	295.11 (211.40)	90.17 (125.11)	385.29 (272.09)	9.26 (2.69)	7.04 (0.78)	20.86 (1.86)	178.71 (128.66)	3.43 (0.73)	0.14 (0.38)
	Feb 24–Mar 2	68.56 (99.98)	126.25 (139.01)	194.81 (194.19)	153.73 (202.33)	348.53 (305.60)	8.02 (2.83)	7.97 (1.20)	6.29 (3.45)	69.59 (51.96)	3.79 (0.91)	0.14 (0.38)
	May 4–10	123.18 (116.69)	188.54 (154.76)	311.72 (215.99)	107.57 (134.16)	419.29 (282.63)	8.98 (2.74)	7.42 (1.14)	21.43 (5.16)	36.06 (11.76)	3.01 (0.19)	0.57 (0.54)
2015–2016 cohort ^d^	Sep 14–20	124.21 (130.44)	205.28 (158.53)	329.49 (227.73)	101.97 (135.78)	431.46 (295.40)	8.89 (2.73)	7.03 (0.73)	27.00 (0.82)	89.24 (43.36)	3.00 (0.00)	0.14 (0.38)
	May 2–8	116.60 (126.69)	175.83 (140.89)	292.43 (211.47)	113.40 (160.89)	405.84 (284.91)	8.66 (2.80)	7.34 (1.01)	25.57 (2.44)	43.43 (17.75)	3.14 (0.24)	0.29 (0.49)
2016–2017 cohort ^e^	Nov 21–27	112.62 (101.77)	177.41 (144.48)	290.03 (194.63)	84.29 (125.30)	374.32 (255.54)	9.45 (2.86)	7.12 (0.93)	5.57 (1.72)	84.43 (96.55)	3.29 (0.06)	0.00 (1.00)
	May 15–21	99.05 (122.52)	166.58 (141.79)	265.63 (208.96)	100.93 (139.56)	366.56 (276.47)	9.22 (2.90)	7.19 (0.98)	20.86 (5.18)	57.71 (21.04)	3.15 (0.24)	0.14 (0.38)
2017–2018 cohort ^f^	Nov 13–19	129.40 (105.09)	199.29 (145.71)	328.69 (205.34)	139.52 (146.50)	468.21 (278.78)	9.15 (2.81)	7.00 (0.95)	2.29 (2.21)	37.43 (27.73)	3.71 (0.95	0.00 (0.00)
	Apr 30-May 6	130.41 (126.73)	190.57 (154.66)	320.99 (227.55)	196.37 (281.07)	517.36 (394.65)	8.49 (2.89)	7.12 (1.13)	19.43 (1.99)	34.29 (23.78)	3.35 (0.56)	0.00 (0.00)

Notes: Air pollution data were collected in the following periods: ^a^ 2012–2013 cohort: 17–27 September 2012, 29 October–8 November 2012, 25 February–8 March 2013, 6–16 May 2013; ^b^ 2013–2014 cohort: 9–15 December 2013, 5–11 May 2014; ^c^ 2014–2015 cohort: 6–12 October 2014, 24 February–2 March 2015, 4–10 May 2015; ^d^ 2015–2016 cohort: 14–20 September 2015, 2–8 May 2016. ^e^ 2016–2017 cohort: 21–27 November 2016, 15–21 May 2017. ^f^ 2017–2018 cohort: 13–19 November 2017, 30 April–6 May. PA: physical activity, SB: sedentary behavior, VPA: vigorous physical activity, MPA: moderate physical activity, MVPA: moderate to vigorous physical activity.

**Table 3:** Estimated effects of temperature on individual-level sleeping outcomes in the past week per day by gender.

Dependent Variable	Male Only		Female Only		Total	
	Coefficient (95% CI)	# Observations (Participants)	Coefficient (95% CI)	Observations (Participants)	Coefficient (95% CI)	Observations (Participants)
*VPA*						
	0.70 *** (0.49, 0.90)	23,011 (9490)	0.59 *** (0.30, 0.88)	10,896 (4446)	0.66 *** (0.49, 0.82)	33,923 (13,802)
*MPA*						
	0.49 ** (0.20, 0.77)	23,011 (9490)	0.68 ** (0.25, 1.11)	10,896 (4446)	0.56 *** (0.32, 0.79)	33,923 (13,802)
*MVPA*						
	1.18 *** (0.80, 1.57)	23,011 (9490)	1.27 *** (0.71, 1.83)	10,896 (4446)	1.21 *** (0.90, 1.53)	33,923 (13,802)
*Walk*						
Walking in last week (min/week)	0.55 *** (0.27, 0.82)	23,011 (9490)	0.53 * (0.10, 0.96)	10,896 (4446)	0.55 *** (0.31, 0.78)	33,923 (13,802)
*Total PA*						
PA in last week (min/week)	1.73 *** (1.23, 2.23)	23,011 (9490)	1.80 *** (1.07, 2.53)	10,896 (4446)	1.76 *** (1.1.35, 2.17)	33,923 (13,802)
*SB*						
Siting in last week (min/week)	−0.63 (−2.62, 1.35)	23,011 (9490)	1.48 (−1.68, 4.65)	10,896 (4446)	−0.25 (−1.93, 1.43)	33,923 (13,802)
*Sleep*						
(min/week)	−1.36 *** (−1.95, −0.77)	22,876 (9486)	−2.38 *** (−3.27, −1.48)	10,834 (4445)	−1.60 *** (−2.09, −1.11)	33,726 (13,799)

Notes: Separate individual fixed-effect regressions were performed to estimate the effects of air pollution concentrations on samples stratified by sex. Models adjusted for all time-variant individual characteristics are listed in Table 1 (i.e., age, BMI, smoking status, drinking status, self-rated physical health, and self-rated mental health), and environmental variables are listed in Table 2 (average air quality index and percentage of rainy days in the last week). * *p* < 0.05; ** *p* < 0.01; *** *p* < 0.001. PA: physical activity, SB: sedentary behavior, VPA: vigorous physical activity, MPA: moderate physical activity, MVPA: moderate to vigorous physical activity.

## Data Availability

The datasets generated and/or analyzed during the current study are not publicly available due to confidentiality but are available from the corresponding author on reasonable request.

## Abbreviations

PA	physical activity
SB	sedentary behavior
IPAQ	International Physical Activity Questionnaire
CPSQI	The Pittsburgh Sleep Quality Index
VPA	vigorous physical activity
MVPA	moderate to vigorous physical activity
BMI	body mass index
